# Supplementary material for: From sequence to enzyme mechanism using multi-label machine learning
Source: BMC Bioinformatics. 2014 May 19;15:150. doi: 10.1186/1471-2105-15-150 (PMC4229970; doi:10.1186/1471-2105-15-150)
Supplement: Additional file 2 — Java code of ml2db. Additional file ml2db_code.tar.gz contains the Java source code to run the multi-label machine learning experiments and save the results to database. The code’s Javadoc is included. [file 1471-2105-15-150-S2.zip › additional file 2/ml2db/ml2db/doc/mulan/evaluation/Evaluation.html]

Evaluation


JavaScript is disabled on your browser.


- Overview
- Package
- Class
- Use
- Tree
- Deprecated
- Index
- Help

- Prev Class
- Next Class

- Frames
- No Frames

- All Classes

- Summary:
- Nested |
- Field |
- Constr |
- Method

- Detail:
- Field |
- Constr |
- Method


mulan.evaluation

## Class Evaluation

- java.lang.Object
- - mulan.evaluation.Evaluation

- ---

    

  ```
  public class Evaluation
  extends java.lang.Object
  ```

  Simple class that includes a list of evaluation measures returned from a
  call to the static methods of `Evaluator` for evaluation purposes.

  Version:
  :   2010.11.05

  Author:
  :   Jozef Vilcek, Grigorios Tsoumakas

  See Also:
  :   `Evaluator`

- - ### Constructor Summary

    Constructors

    Constructor and Description

    `Evaluation(java.util.List<Measure> someMeasures,
    MultiLabelInstances data)`

    Creates a new evaluation object by deep copying the measure objects that
    are given as parameters
  - ### Method Summary

    Methods

    | Modifier and Type | Method and Description |
    | --- | --- |
    | `java.util.List<Measure>` | `getMeasures()` Returns the evaluation measures |
    | `java.lang.String` | `toCSV()` Returns a CSV representation of the calculated measures |
    | `java.lang.String` | `toString()` Returns a string with the results of the evaluation |

    - ### Methods inherited from class java.lang.Object

      `equals, getClass, hashCode, notify, notifyAll, wait, wait, wait`

- - ### Constructor Detail


    - #### Evaluation

      ```
      public Evaluation(java.util.List<Measure> someMeasures,
                MultiLabelInstances data)
                 throws java.lang.Exception
      ```

      Creates a new evaluation object by deep copying the measure objects that
      are given as parameters

      Parameters:
      :   `someMeasures` - calculated measures
      :   `data` - the evaluation data used for obtaining label names for per
          outputting per label values of macro average measures

      Throws:
      :   `java.lang.Exception`
  - ### Method Detail


    - #### toString

      ```
      public java.lang.String toString()
      ```

      Returns a string with the results of the evaluation

      **Overrides:**
      :   `toString` in class `java.lang.Object`

      Returns:
      :   a string with the results of the evaluation


    - #### toCSV

      ```
      public java.lang.String toCSV()
      ```

      Returns a CSV representation of the calculated measures

      Returns:
      :   the CSV representation of the calculated measures


    - #### getMeasures

      ```
      public java.util.List<Measure> getMeasures()
      ```

      Returns the evaluation measures

      Returns:
      :   the evaluation measures


- Overview
- Package
- Class
- Use
- Tree
- Deprecated
- Index
- Help

- Prev Class
- Next Class

- Frames
- No Frames

- All Classes

- Summary:
- Nested |
- Field |
- Constr |
- Method

- Detail:
- Field |
- Constr |
- Method
